# Epidemiology of soil-transmitted helminth infections and the differential effect of treatment on the distribution of helminth species in rural areas of Gabon

**DOI:** 10.1186/s41182-023-00567-z

**Published:** 2024-01-02

**Authors:** Jean Ronald Edoa, Bayodé Roméo Adégbitè, Yabo Josiane Honkpéhèdji, Jeannot Fréjus Zinsou, Stravensky Térence Boussougou-Sambe, Tamirat Gebru Woldearegai, Benjamin Mordmüller, Ayola Akim Adegnika, Jean Claude Dejon‑Agobé

**Affiliations:** 1 Centre de Recherches Médicales de Lambaréné, P.O. Box 242, Lambaréné, Gabon; 2 Center of Tropical Medicine and Travel Medicine, Department of Infectious Diseases, Division of Internal Medicine, Amsterdam University Medical Centers, Location AMC, Amsterdam Public Health, Amsterdam Infection and Immunity, University of Amsterdam, Amsterdam, The Netherlands; 3 Fondation Pour la Recherche Scientifique, ISBA, P.O. Box 88, Cotonou, Bénin; 4 Department of Parasitology, Leiden University Medical Center, 2333 ZA Leiden, The Netherlands; 5 Institut für Tropenmedizin, Universitätsklinikum Tübingen, Wilhelmstraße 27, 72074 Tübingen, Germany; 6 German Center for Infection Research, Tübingen, Germany; 7 Department of Medical Microbiology, Radboud University Medical Center, Nijmegen, The Netherlands

**Keywords:** Anthelminthic treatment, *Ascaris lumbricoides*, Epidemiology, Gabon, Hookworm, Incidence, Prevalence, *Trichuris trichiura*

## Abstract

**Background:**

Soil-transmitted helminth (STH) infections are a public health concern in endemic areas. For efficient control, the epidemiology of the disease needs to be monitored. This report assesses the prevalence, incidence, post-treatment infection (PTI) rate, and risk factors for STH infections in two rural areas of Gabon.

**Method:**

In this longitudinal and prospective study, participants aged six to 30 years from the vicinity of Lambaréné and selected households using a simple randomization process were included and followed in two consecutive periods of six and nine months. Stool samples were obtained at the beginning and the end of each follow-up phase (FUP). The Kato-Katz technique was used for the detection of STH eggs, while the Harada-Mori technique and coproculture were used for the detection of larvae in stool processed within a maximum of four hours of collection. Prevalence was determined at the three main time points of the study, incidence was assessed during the two study phases, and PTI was defined as an infection detected nine months post-treatment.

**Results:**

A total of 262 participants were included. The overall prevalence of STH infections was 42% (95%CI: 34–50) and 44% (95%CI: 37–51) at baseline for the six and nine month FUPs, respectively. *Trichuris trichiura* was the most prevalent species at each time point of assessment. The cumulative incidence of STH at the 6- and 9-month follow-ups was 18% (95%CI: 12–27) and 35% (95%CI: 27–43), respectively, while the incidence rates were 41 (95%CI: 28–55) and 56 (95%CI: 46–67) per 100 person-years, respectively. The PTI rates at the 9-month follow-up for *T. trichiura*, hookworm, and *Ascaris lumbricoides* were 58% (95%CI: 41–74), 31% (95%CI: 11–59) and 18% (95%CI: 5–40), respectively. The STH infection intensity was generally light.

**Conclusion:**

The prevalence level of STH infection is moderate in the vicinity of Lambaréné, with *T. trichiura* being the most prevalent species. Our results reveal a rapid spread of the disease in the population mainly following intervention, particularly for trichuriasis, and therefore call for the full implementation of the World Health Organization’s recommendations in the area.

*Trial registration* clinicaltrials.gov Identifier NCT02769013. Registered 21 April 2016, https://clinicaltrials.gov/study/NCT02769013

**Supplementary Information:**

The online version contains supplementary material available at 10.1186/s41182-023-00567-z.

## Introduction

Soil-transmitted helminths (STHs) are helminths transmitted to humans by contaminated soil. The most prevalent STH species include *Ascaris lumbricoides*, *Trichuris trichiura, Strongyloides stercoralis,* and hookworms (*Necator americanus* and *Ancylostoma duodenale*). Transmission occurs when eggs of the parasite are present in human faeces and contaminate the soil. Two modes of transmission can be described: first, by active skin penetration of larvae for hookworm and *S. stercoralis*, although the latest autoinfection is possible and therefore very often leads to chronic infections, and second, through ingestion of eggs in contaminated food or water for *A. lumbricoides* and *T. trichiura*.

STH infections are among the most common infections worldwide and represent a significant public health threat. The World Health Organization (WHO) reported in 2020 an estimated 1.5 billion people infected with STH worldwide, with over 267 million preschool-age children and over 568 million school-age children living in areas where these parasites are transmitted [1]. These infections are widely distributed in tropical and subtropical regions due to a combination of factors, such as living conditions and climatic influences. Indeed, the highest burden of STH infections is observed in Southeast Asia and sub-Saharan Africa, where warm and humid climates provide favourable conditions for the survival of STH parasites [1] while poor living conditions, inadequate access to safe water, and a lack of proper sanitation facilities further contribute to their transmission [2]. Poorest and most deprived communities consequently bear the high burden of the disease [2].

STH infections commonly lead to disease with relatively mild symptoms. However, the severity of the infection and associated morbidity can vary depending on the intensity of the infection. In addition to mild symptoms, more severe forms of the infection can occur, resulting in various clinical manifestations and long-term sequelae. These include general malaise, fatigue, intestinal manifestations (diarrhoea and abdominal pain), anaemia, growth impairment, intellectual retardation, and cognitive and educational deficits [3]. Benzimidazole drugs are effective for treating of STH infections. In addition, albendazole (ABZ) and mebendazole, also used in mass drug administration (MDA) campaigns for STH control, have indeed demonstrated high efficacy (with a more than 95% egg reduction rate) against *A. lumbricoides* and hookworm infections [4] but low efficacy against *T. trichiura* infection, with a cure rate of approximately 50% [4–6]. To improve the efficacy against *T. trichiura*, repeated administration of ABZ has been suggested with a better outcome [4]. Despite the satisfactory efficacy of these drugs and when administered repeatedly for *T. trichiura* [4, 7], a high risk of reinfection is observed [8, 9], probably because of early re-exposure. Therefore, for effective control of STH infections, it is crucial to provide not only anthelmintic treatment for the reduction of disease morbidity, but also implement comprehensive interventions to prevent exposure to the disease [10, 11].

Gabon is a Central African country with approximately 2.3 million inhabitants [12]. The country is known to be endemic for STH infections [13] where rural area is one of the risk factors for the disease as reported by Mintsa Nguéma et al. [14], and with children particularly exposed to the disease. Indeed, the WHO stated in 2018 that 483,207 children, including 156,713 preschool aged children and 326,494 school-aged children, required preventive chemotherapy in the country [15]. Despite the evidence of their presence in Gabon, epidemiological data on STH are sparse, highlighting the necessity to characterize the epidemiological profile of the disease in the country. In general, prevalence and risk factors are very often reported as epidemiological data of STHs. To the best of our knowledge, no study has addressed the dynamics and spread of these infections in Gabon. In the present analysis, we report the prevalence and dynamics of STH infections before and after treatment, and the incidence of STH infections among children and young adults living in rural Gabon.

## Materials and methods

### Study design

The present analysis is a subanalysis of a longitudinal, prospective and assessor blind study designed to assess the effect of pre- and post-treatment of schistosomiasis with praziquantel on malaria transmission.

### Study area and study population

The study was conducted in the vicinity areas (Zilé-PK area and Bindo village) of Lambaréné. Those rural areas are known to be endemic to STH [4, 16]. They have inadequate environmental sanitation and poor hygiene [17]. The Zilé-PK area is a set of villages at point km 14 along the N1 national road southeast of Lambaréné. The area is characterized by the absence of pipe water with few public pumps which are not always functional. Small tributaries of the Ogooué River and wells are therefore the main water source for the population for their daily activities. Houses are mainly wooden and usually have an earthen floor and a pit latrine aside. The area has two primary schools and two dispensaries. In contrast to the Zilé-PK area, Bindo village is a remote area located 65 km away from Lambaréné. It is mainly built around a commercial palm plantation. The village is populated by approximately 1000 inhabitants, mainly workers of the palm plantation and their families. Bindo is characterized by the presence of electricity and two public taps supplied directly by the Ogooué River through a pipeline. Tributaries of the Ogooué River and wells are also used as water sources by the population. Houses are mainly made of concrete and have concrete floors. There is one primary school and one dispensary. To be eligible to participate in the present study, volunteers should be apparently healthy, aged from six to 30 years, and living in the study area.

### Sample size consideration

The first objective of this analysis was to determine at baseline the prevalence of any STH infection in the community where the study population was randomly selected. From the main study, we were able to include in the analysis 268 participants with stool results available. Using the formula of sample size calculation for a cross-sectional study [18] and considering a previous 31% prevalence of any STH infection reported in the area a decade ago [19], the sample size of 268 gives us a 95% chance (confidence level) that the real value of STH prevalence in the community is within ± 5. 54% (margin of error) of the measure value.

### Sampling procedure

The selection of participants in the main study was performed in two steps: (i) census of all the houses present in the study area, and (ii) household selection using a simple randomization procedure. For each household selected, inhabitants aged 6–30 years old who agreed to participate in the study underwent the informed consent process and eligibility criteria assessment.

### Study procedure

**Fig. 1:**
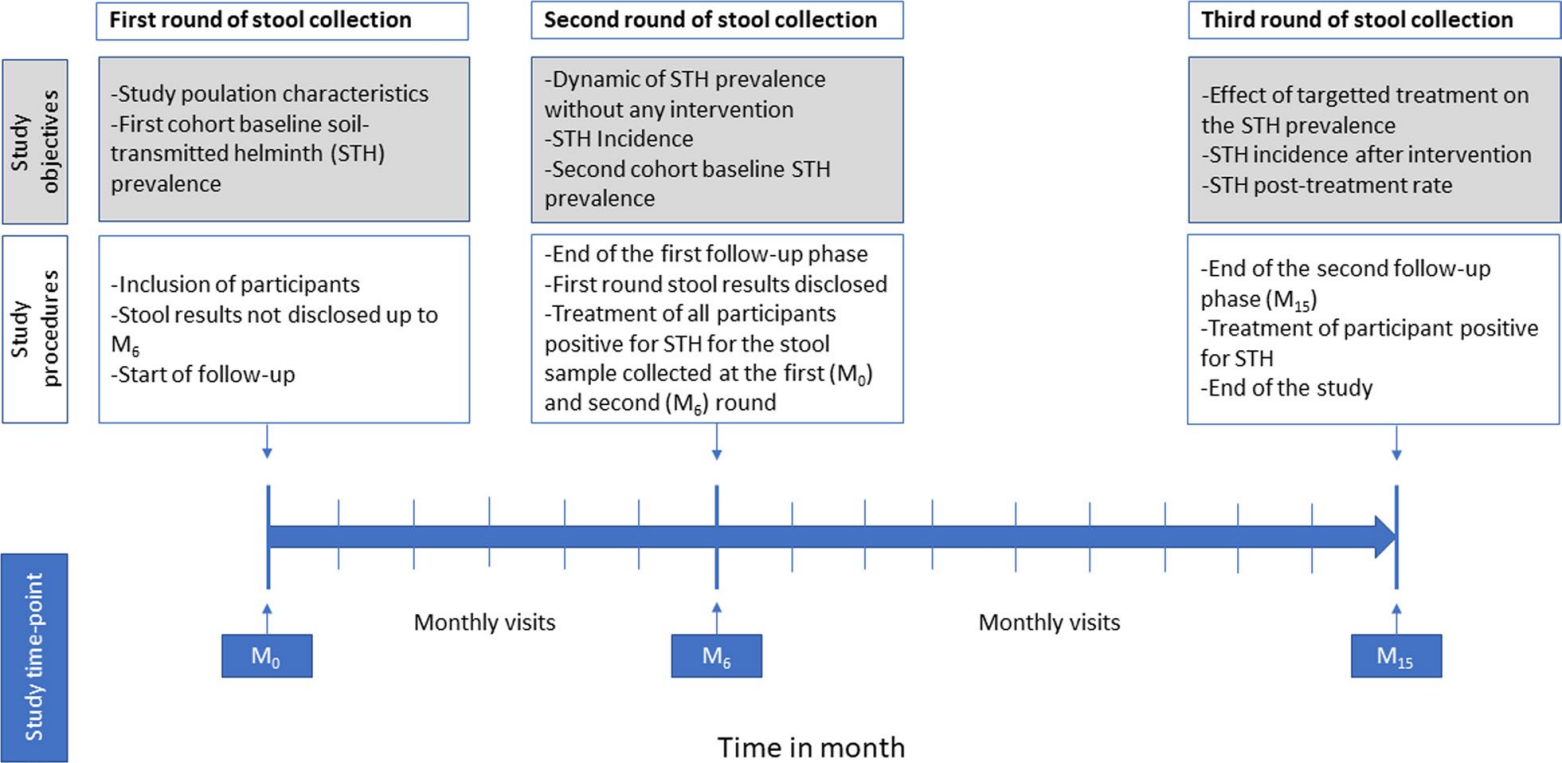
Study design

The study was conducted from June 2016 to November 2018. As previously described [20], the follow-up (FU) consisted of monthly visits to the participants’ homes by the study team during which participants’ health was checked and intake of medications was investigated and recorded. As depicted in Fig. 1, all participants were seen in the study at the M_0_ visit during which baseline information was collected. Participants were followed in two phases of six and nine months, respectively, with three different time points where stool samples were collected: at baseline (M_0_), at the end of the first 6-month FU phase (M_6_), which was also considered the beginning of the second study phase, and at the end of the second 9-month FU phase (M_15_). In the frame of this analysis, only participants from the main study [20] who provided at least one stool sample at any of these three time points were considered. We therefore considered in the first follow-up phase (FUP) all participants who provided a stool sample at inclusion (M_0_), while in the second FUP, we considered participants who provided a stool sample at M_6_ and/or at M_15_. Stool collection at M_6_ comprised participants from the first FUP and those who provided stool samples for the first time at that time point. The results of stool samples collected at M_0_ were not released until the end of the first FU period (M_6_) unless the participant had helminthiasis related symptoms anytime during the FUP. This allowed assessment of the dynamic of the infection without intervention. All participants positive at M_6_ and those with positive stool samples from M_0_ were treated for STH infection before starting the second FUP which lasted nine months (from M_6_ to M_15_), equating to a total of 15 months of FU for both phases. Stool samples collected at M_15_ were immediately tested and when positive, the participants were treated. For any medical concern occurring between the monthly visits, participants were invited to come to the research centre to receive medical care. In the case of gastrointestinal symptoms, a stool sample was collected and examined for the presence of STH eggs and/or larvae, and the participant was treated if infected. STH infection was treated with 400 mg of ABZ (DAPMéd-Africa) once a day for three consecutive days [4]. All treatments were administered under the observation of a member of the study team.

### Stool sample collection procedure

Stool samples were collected by the participant himself. A single stool sample was requested from each participant at each time point of sample collection. A plastic container was provided to each participant along with clear instructions for stool collection. The collection process was explained to each participant, and for young children, instructions were given to the parents who were in charge of assisting the child. After receiving the plastic container, the participant was requested to provide the stool sample in the morning of the next day. In the morning of sample collection, the stool sample was picked up from the participant by a member of the research team between 8 a.m. and 10 a.m. and immediately placed in a temperature-controlled cooler to maintain appropriate storage conditions. After collection, the samples were transported within a maximum of four hours to the parasitological laboratory where they were immediately processed.

### Laboratory examination

For the diagnosis of STH infections, we performed microscopy techniques using the fresh stool samples we collected. To increase the sensitivity of the method, particularly for hookworm and *Strongyloides stercoralis* infections, we performed parallel testing using three tests: Kato-Katz, Harada-Mori technique, and coproculture [21]. We used the Kato-Katz technique for the detection of *A. lumbricoides*, *T. trichiura* and hookworm eggs, while the Harada-Mori technique and coproculture were used for the detection of *S. stercoralis* and hookworm larvae.

### Case definitions

A participant was considered infected with STH if the stool sample was positive for the presence of at least one STH egg or larva, irrespective of the STH species. Otherwise, the participant was considered negative. When positive on at least one time point of stool collection, a participant was allocated to the STH-positive group, while those who remained negative at all stool collection time points were allocated to the STH-negative study group. Except for *S. stercoralis* infection for which it is not possible to have the egg count, the intensity of the respective STH infection was defined as light, moderate or heavy according to the threshold of the egg count of each STH species (see Additional file 1: Table S1).

### Statistical consideration

The participants’ sociodemographic data were collected at baseline, including age, gender and location. Their STH status was assessed at the three different study time-points of stool collection: M_0_, M_6_ and M_15_ and, if clinically indicated. Data were collected using a paper-based case report form and digitalized using REDCap hosted at the Centre de Recherches Médicales de Lambaréné (CERMEL) [22]. The clean database was imported into R software (version 4.0.2) for analysis. The prevalence of STH infections was calculated by dividing the number of positive cases by the total number of participants tested. This was done for any STH as well as for the respective species. The incidence was estimated at the end of both follow-up phases among participants who provided a stool sample and were previously negative for the respective STH species (first follow-up and second follow-up) or treated (second follow-up phase). The cumulative incidence (or incidence proportion) and the incidence rate expressed in person-years were therefore obtained by dividing the number of new positive cases occurring between M_0_ to M_6_ and M_6_ to M_15_ by the total number of participants tested during the same period or by the total time of at risk of infection, respectively. Time at risk for participants negative for STH at M_0_ was defined as six months for those who remained negative at M_6_ and as three months for those positive, assuming that for these last ones, the infection occurred halfway between M_0_ and M_6_. In the same manner, time at risk during the second follow-up phase (FUP) was defined as nine months for participants found negative at the end of the FUP (M_15_), or 4.5 months for those found positive. The post-treatment infection (PTI) rate was defined as the number of STH infections observed during the second FUP and particularly at M_15_ among those treated at M_6_. Categorical variables were summarized as proportions and 95% confidence intervals (CI), while continuous variables were summarized by means and standard deviations (SD) when normally distributed. The dynamic of STH infections was assessed by comparing the prevalence from inclusion to the end of the FU for each study phase. Otherwise, the variable was summarized as median and interquartile range (IQR). Study groups were compared using the Chi square test while incidences between both study phases were compared using the *binom.test()* function from the *stats* package in R. Logistic regression was performed to assess factors associated with STH infection. A double-sided *p value* less than 5% was considered statistically significant.

## Results

### Study population characteristics and study groups

As presented in Table 1, a total of 262 participants were included in the analysis. The mean (SD) age was 12.1 (SD = 4.8) years, with a female-to-male sex ratio of 0.98. Eighty-three percent (218/262) of the study population came from the Zilé-PK area. Out of the 262 participants, 138 (53%) were infected with STH and were included in the STH-positive (STH^+^) study group while their counterparts negative for STH infection were included in the STH-negative (STH^−^) study group. There was no statistically significant difference in age between the study groups (*p* value = 0.24). However, in the STH-positive group, there is a trend for males to be overrepresented compared to females (56% vs. 44%, *p* value = 0.08). Regarding participant location, a statistically significant association was found in both study groups, as participants predominantly came from the Zilé-PK area rather than the Bindo village (*p* value = 0.02).

**Table 1:** Characteristics of study population and study groups. The STH-positive study group included participants with at least one stool sample positive for STH eggs and/or larvae over the study course

	Overall population			STH-negative study group		STH-positive study group		*p*-value
	n	%	95% CI	n	%	n	%	
Total	262	–	–	124	47.3	138	52.7	
Age (mean, SD)	12.1	(4.8)	–	11.8	(4.1)	11.2	(3.8)	0.24
Sex								0.08
Female	130	49.6	43.4–55.8	69	55.6	61	44.2	
Male	132	50.4	44.2–56.6	55	44.4	77	55.8	
Ratio (F/M)	0.98	–	–	1.32	–	0.77	–	
Address								0.02
Bindo	44	16.8	12.5–21.9	22	17.7	22	15.9	
Zilé-PK area	218	83.2	78.1–87.5	102	82.3	116	84.1	

### Stool sample collection flow and follow-up outcomes

**Fig. 2:**
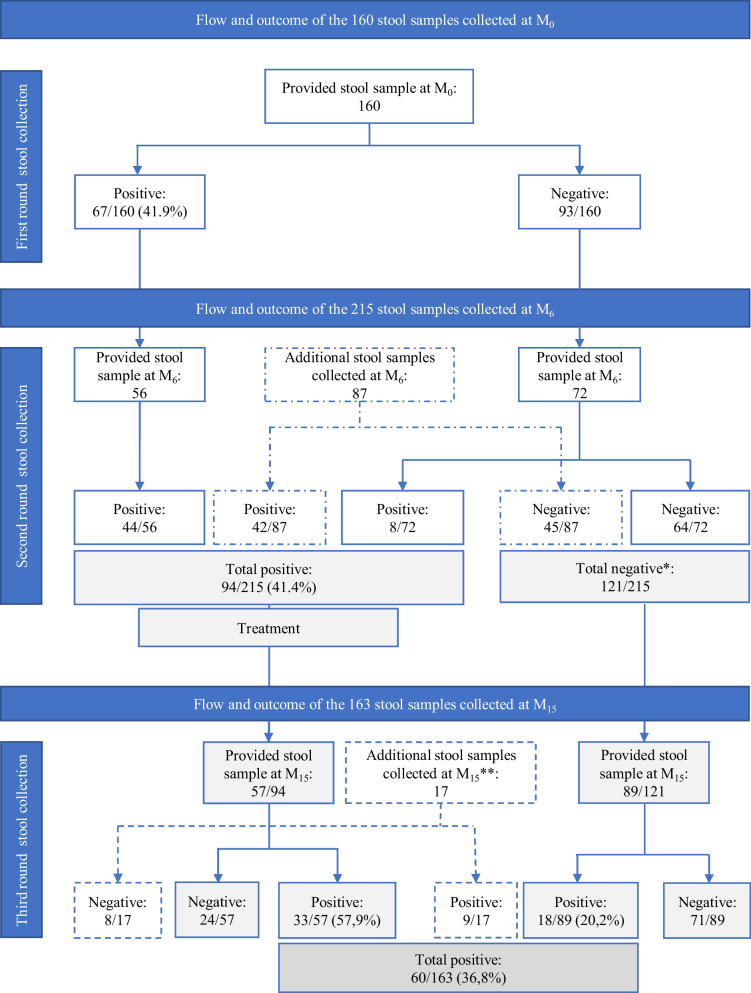
Stool collection flow and STH participants status at different study time-points. * + 12 participants negative out of the 56 positive at the first round and present at the second round. **Three out of the 17 participants provided stool sample also at M_0_ visit

Of the study participants followed over the 15 months of the study course, 262 provided a stool sample at least at one of the three study time-points where a stool sample was requested; M_0_, M_6_, and M_15_. As depicted in Fig. 2, 160 participants provided stool sample at M_0_, and constituted the first cohort. Among them, 67/160 (42%) were positive for any STH at M_0_. The second round of stool collection was carried out at 6 months (M_6_) of the participant FU, where 215 participants provided stool samples and thus constituted the second cohort. Among them, 128 participants who completed the first FU phase and 87 who provided stool sample for the first time at that time-point (M_6_). Of those 215 participants, 94 (41%) were tested positive for any STH, all treated, and FU for nine months (second FU phase). At the end of the second FU phase, 146 (68%) out of the 215 included provided stool samples. In addition to them, 14 participants who provided stool sample for the first time and three who provided stool samples at M_0_ but not at M_6_ were included, yielding a total of 163 stool samples collected at M_15_. Of them, 60/163 (37%) samples were positive for any STH. No stool sample was collected outside of the three time points of stool collection since no participant reported symptoms related to helminthiasis during the study course.

### STH prevalence and prevalence dynamic over the study course

As described in Table 2, the overall prevalence of any STH infections was 42% (67/160; 95%CI: 34–50) and 44% (94/215; 95%CI: 37–51) at inclusion for both cohorts (M_0_ and M_6_), respectively. At both time points, *T. trichiura* was the most prevalent STH species with 29% (46/160; 95%CI: 22–36) and 31% (66/215; 95%CI: 25–37) prevalence, respectively. Except for *S. stercoralis* infection, for which we did not determine the intensity of infection, all cases of Hookworm and *T. trichiura* infections were of light intensity, while 6% (5/80) of *A. lumbricoides* infections were of moderate intensity, and the remaining 94% were of light intensity. As presented in Table 2, no difference (*p* value = 0.92) was observed in the prevalence level for any STH infection from M_0_ to M_6_ among participants present at both time points, while a slight but not statistically significant decrease was observed in the prevalence level from M_6_ to M_15_ among participants included in the second FUP either for any STHs (44% vs. 35%, *p*-value = 0.12), or for *A. lumbricoides* (17% vs. 11%, *p*-value = 0.17), *T. trichiura* (31% vs. 24%, *p*-value = 0.20), and hookworm (13% vs. 9%, *p value* = 0.30). The decrease in the prevalence observed was statistically significant only for *S. stercoralis* (11%, 95%CI: 7–16 vs. 2%, 95%CI: 0.4–6, *p*-value = 0.004). Furthermore, among the participants diagnosed with ascariasis at M_0_, 9 (45%) out of 20 were found to be negative for ascariasis at M_6_. Similarly, 6 (15%) out of 40, 7 (50%) out of 14, and 5 (62%) out of 8 participants tested negative at M_0_ for trichuriasis, hookworm infection, and strongyloidiasis, respectively, were found to be negative for the respective species at M6.

**Table 2:** Prevalence of soil-transmitted helminths (STHs) assessed at inclusion and at the end of each follow-up phase

	First follow-up						*p*-value	Second follow-up						*p*-value
	Inclusion (M_0_), *N* = 160			Six-month follow-up (M_6_), *N* = 128				Inclusion (M_6_), *N* = 215			Nine-month follow-up (M_15_), *N* = 146			
	n	%	95% CI	n	%	95% CI		n	%	95% CI	n	%	95% CI	
Any STH	67	41.9	34.1–49.9	52	40.6	32.0–49.7	0.92	94	43.7	37.0–50.6	51	34.9	27.2–43.2	0.12
* Ascaris lumbricoides*	26	16.2	10.9–22.9	18	14.1	8.5–21.3	0.73	36	16.7	12.0–22.4	16	11.0	6.4–17.2	0.17
* Trichuris trichiura*	46	28.7	21.9–36.4	40	31.2	23.3–40.0	0.74	66	30.7	24.6–37.3	35	24.0	17.3–31.7	0.20
Hookworm	15	9.4	5.3–15.0	14	10.9	6.1–17.7	0.81	28	13.0	8.8–13.3	13	8.9	4.8–14.7	0.30
* Strongyloides stercoralis*	10	6.2	3.0–11.2	12	9.4	4.9–15.8	0.44	23	10.7	6.9–15.6	3	2.0	0.4–5.9	0.004

n: number of positive participants

### Incidence of STH at 6- and 9-month follow-up

As presented in Table 3, the incidence proportions of any STH infection at 6 months (from M_0_) and 9 months (from M_6_) follow-up were 18% (23/124; 95%CI: 12–27) and 35% (51/146; 95%CI: 27–43), respectively, while the incidence rates were 41 (95%CI: 28–55) and 56 (95%CI: 46–67) per 100 person-years, respectively. Considering the STH species, no significant difference was observed either in the incidence proportion or in the incidence rate between the STH species during the first FU phase (95%CI overlapping), while during the second FU phase, *T. trichiura* had the highest incidence and *S. stercoralis* had the lowest incidence. Comparing the two study FU phases, a significant difference was observed only for *T. trichiura* (*p* value < 0.001) with an increased incidence observed during the second FU phase, as compared to the first FU phase for both incidence proportion (24% vs. 7%) and incidence rate (36.3 vs. 14.6 per 100 person-years). Only a non-statistically significant increasing trend in the incidence level was observed for *A. lumbricoides* (*p*-value = 0.09), while a non-statistically significant decrease in the incidence level was observed for *S. stercoralis* (*p*-value = 0.15). No statistically significant difference was observed for hookworm infection in terms of both incidence proportion and incidence rate (*p*-value = 0.26).

**Table 3:** Incidence proportion (I_P_) and incidence rate (I_R_) of STH species over the study course

	First follow-up phase, 6 months							Second follow-up phase, 9 months							*p*-value*
	*N*	*n*	I_P_ (%)	95%CI	E.T	I_R_	95%CI	*N*	*n*	I_P_ (%)	95%CI	E.T	I_R_	95%CI	
Any STH species	124	23	18.5	12.3–26.7	56.2	40.9	28.0–54.8	146	51	34.9	27.4–43.3	90.4	56.4	45.6–66.8	0.001
*Ascaris lumbricoides*	105	7	6.7	2.9–13.7	50.7	13.8	5.7–26.4	146	16	11.0	6.6–17.5	103.5	15.5	9.1–23.9	0.09
*Trichuris trichiura*	85	6	7.1	2.9–15.3	41.0	14.6	5.6–29.2	146	35	24.0	17.5–31.9	96.4	36.3	26.8–46.7	< 0.001
*Hookworm*	109	7	6.4	2.8–13.2	52.7	13.3	5.5–25.4	146	13	8.9	5.0–15.0	104.6	12.4	6.8–20.3	0.26
*Strongyloides stercoralis*	115	9	7.8	3.9–14.7	55.2	16.3	7.7–28.7	146	3	2.0	0.5–6.3	108.4	2.8	0.6–7.9	0.15

*p-value using binom.test of stats r package comparing the Ip and I_R_ at the two time points

N: Total number of participants considered as negative at the beginning of the follow-up phase and for the respective species; n: Total number of positive participants; E.T: Exposure time expressed in 100 person-year

### STH infection rate at 9 months post-treatment

As shown in Fig. 2, of the 94 participants found positive for any STH at the beginning of the second FU phase (M_6_) and treated, 57 were present at the end of the 9-month follow-up (M_15_). Of them, 33 (58%; 95%CI: 44–71) were found to be positive for any STH at M_15_ (Table 4). Considering the STH species at M_15_, of the 14 cases of ascariasis treated at M_6_, ten (71%; 95%CI: 42–92) converted to STH-negative while four (29%; 95%CI: 8–58) were still positive for ascariasis. In addition, five new ascariasis cases were observed. Similarly, of the 25 participants treated for trichuriasis at M_6_, 22 (88%; 95%CI: 69–97) had the same infection and only three (12%; 95%CI: 2.5–31.2) participants converted to STH-negative. Four trichuriasis cases observed at M_15_ were new. Of the 11 participants treated for hookworm infection, six (54%; 95%CI: 23–83) converted to STH-negative while five (45%; 95%CI: 17–77) were still infected. Three cases of hookworm infection observed at M_15_ were new. With regard to *S. stercoralis* infection, the only case observed at M_15_ was a new case.

**Table 4:** Nine months post-treatment infection rate among the 57 study participants assessed during the second follow-up phase of the study

	Second follow-up study phase			
	*N*	*n*	%	95% CI
Any STH	33	25	75.8	57.7–88.7
*Ascaris lumbricoides*	14	4	28.6	8.4–58.1
*Trichuris trichiura*	25	22	88.0	68.8–97.4
Hookworm	11	5	45.4	16.7–76.6
*Strongyloides stercoralis*	8	0	0.0	–

N: Number of participants positive for the respective STH species and treated at the beginning of the second follow-up study phase and present at the end of the follow-up phase; n: number of participants positive at the end of the second follow-up phase among the N

### Factors associated with STH infection

We investigated age, sex and location as potential factors associated with exposure to STH parasites and being in the positive study group. Similar to the univariate analysis results (Table 5), we found at the multivariate analysis level an association between *A. lumbricoides* infection status and age (*p*-value = 0.001), and between hookworm infection status and age (*p*-value = 0.01). Indeed, the odds of having ascariasis decreased as when age increased (aOR = 0.87, 95%CI: 0.79–0.95), while the odds of having hookworm infection increased with age (aOR = 1.11, 95%CI: 1.02–1.21). By investigating the association between gender and STH infection status, we found a relationship between gender and hookworm (*p*-value = 0.01) infection status. Here, males had a trend to have higher odds of being infected with *T. trichiura* (aOR = 1.64; 0.98–2.74) and significantly higher odds of being infected with hookworm (aOR = 2.28; 1.15–4.71). No statistically significant association was found between *S. stercoralis* infection and age (*p*-value = 0.79), gender (*p*-value = 0.16), and location (*p*-value = 0.81).

**Table 5:** Logistic regression analysis assessing the risk of STH infection at any time point of the study with age, gender and location

Variable	Frequency		Crude analysis			Adjusted analysis		
	*n*	%	cOR	95% CI	*p*-value	aOR	95% CI	*p*-value
*Ascaris lumbricoides*								
Age	64	24.4	0.87	0.79–0.95	**0.001**	0.87	0.79–0.95	**0.001**
Gender								
Female	31	23.8	1	–	**0.82**	1	–	**0.87**
Male	33	25.0	1.06	0.60–1.87		1.05	0.59–1.89	
Location								
Bindo	13	29.5	1	–	**0.008**	1	–	**0.17**
PK	51	23.4	0.73	0.36–1.54		0.59	0.28–1.27	
*Trichuris trichiura*								
Age	97	37.0	0.96	0.89–1.02	**0.19**	0.97	0.90–1.03	**0.32**
Gender								
Female	40	30.8	1	–	**0.03**	1	–	**0.06**
Male	57	43.2	1.71	1.03–2.85		1.64	0.98–2.74	
Location								
Bindo	12	27.3	1	–	**0.14**	1	–	**0.26**
PK	85	39.0	1.70	0.85–3.61		1.52	0.74–3.26	
Hookworm								
Age	44	16.8	1.09	1.01–1.17	**0.03**	1.11	1.02–1.21	**0.01**
Gender								
Female	15	11.5	1	–	**0.02**	1	–	**0.02**
Male	29	22.0	2.15	1.11–4.34		2.28	1.15–4.71	
Location								
Bindo	5	11.4	1	–	**0.29**	1	–	**0.20**
PK	39	17.9	1.70	0.68–5.17		1.91	0.73–6.04	
*Strongyloides stercoralis*								
Age			1.00	0.91–1.09	**0.91**	1.01	0.92–1.11	**0.79**
Gender								
Female	13	10.0	1	–	**0.16**	1	–	**0.16**
Male	21	15.9	1.70	0.82–3.64		1.70	0.82–3.66	
Location								
Bindo	5	11.4	1	–	**0.72**	1	–	**0.81**
PK	29	13.3	1.20	0.47–3.69		1.13	0.43–3.55	

## Discussion

This study assessed the distribution and spread of STH infections among schoolchildren and young adults living in two different rural areas endemic for STH parasites. Our results reveal a moderate prevalence of STH infections, with *T. trichiura* and *A. lumbricoides* being the most prevalent. Similarly, we found a moderate rate of infection with the same STH species post-treatment while the STH incidence was relatively high, particularly during months following treatment. Age and gender were found to be associated with the risk of STH infection.

The main indicator we used to assess the distribution of STH infection in our population was the prevalence. We reported a moderate prevalence of any STH infection, with a 42% prevalence found at baseline. A lower prevalence (31%, 95%CI: 27–35) was found in the area in 2012 [19], showing no improvement and ongoing transmission in the local population. This suggests a lack or inefficiency of the STH infection control programme in the study area. Indeed, Gabon has endorsed the WHO recommendation for STH control consisting of the mass drug administration of albendazole among school children. Considering the STH species, *T. trichiura* was the most prevalent species in the area, followed by *A. lumbricoides*. This profile is similar to what has been reported from other areas in Gabon [14, 23] but different from what was reported by Staudacher et al. in Rwanda using Kato-Katz and polymerase chain reaction methods for the diagnosis of STH infection and by Kirwan et al. in Nigeria who used the formol ether concentration technique as a diagnostic method. Both authors reported *A. lumbricoides* as the dominant species [24, 25] in preschool- and school-age children. Although the sensitivity of the diagnostic methods used in different studies may explain some of the observed differences, we hypothesize that the high relative prevalence of *T. trichiura* species we reported could be due to the use in the community of benzimidazole drugs for the treatment of STH infections known to be less effective for trichuriasis [4] and potentially leading to a chronic carriage observed for this infection. Indeed, it has been shown that the peak prevalence of *T. trichiura* and *A. lumbricoides* infection occurs in childhood and drops in persons older than 15 years old for *A. lumbricoides* infection, but not for *T. trichiura* [26, 27]. From our side, we included school age children and young adults, and we found a decreased risk of *A. lumbricoides* infection with age, while the risk of *T. trichiura* infection was similar in different age groups, probably because infection with *T. trichiura* could occur in childhood already in our community and is not efficiently controlled by the benzimidazole (ABZ and mebendazole) used for either the treatment of STH infection cases or in the frame of MDA campaigns.

Assessing the intensity of STH infections, we found light intensity basically for all infections, with only some cases of ascariasis of moderate intensity. In our area where the prevalence of STH infections is moderate, this result could be surprising. However, our result is similar to what is reported by some authors in areas with moderate [28] or even high [29, 30] STH prevalence. As suggested by Meñe et al. [30], we hypothesize that the absence of heavy infection intensity in our community could be explained by frequent deworming of children by their parents, which is a common practice in the country, particularly when the child presents helminth-like symptoms and thus in cases of heavy infection intensity. Such targeted treatment could therefore contribute to the reduction in the intensity of infection cases and probably disease-related morbidity in the community. The contrast between the moderate prevalence and light intensity of infection we found could therefore indicate that such an approach cannot control the risk of infection in the community.

The second indicator we used to assess the presence of STH was the incidence, an indicator of the spread of the disease in the community. Using the incidence proportion, we found an 18% incidence of STH for 6 months of follow-up, indicating that approximately two school children or young adults out of 10 are infected every 6 months, with one of the different STH parasites endemic in the area. More precisely and using the incidence rate, between 28 and 55 per 100 school children and young adults are infected with at least one STH species per year. Applying this incidence to the approximately 12.000 inhabitants aged 6 to 30 years living in the vicinity of Lambaréné as estimated in 2017 (CERMEL-sudesa, 2017. unpublished data), the estimated number of new cases of STH infection irrespective of the species in children and young adults could therefore be between 3300 and 6600 each year. Assessing the incidence of STH species after treatment of the positive cases before the follow-up which could be assimilated to a targeted intervention, we noticed a significant increase in the level of incidence when considering any STH in the cohort, suggesting that our population could be re-infected early after treatment or that the drug we used only induced some reduction in egg production by the parasites. Considering the species, the pattern observed is significant only for *T. trichiura*. Indeed, the incidence of *T. trichiura* was two and a half times higher after treatment than before treatment. As we did not assess the outcome of STH treatment, we hypothesize that this situation could be due to the combination of the high incidence of *T. trichiura* with either the persistence of the infection after treatment, or to an early reinfection. Indeed, *T. trichiura* is reported to occur rapidly after treatment as reviewed by Jia et al. [8]. Indeed, a previous study conducted in the same population years before showed that *T. trichiura* is less sensitive to the treatment protocol we used than the other species. The study reported a dose dependent sensitivity of STHs to ABZ, with *A. lumbricoides* sensitive to one dose of 400 mg, while one dose of 400 mg on two or three consecutive days was necessary to improve the treatment of hookworm and *T. trichiura* [4]. In Indonesia, Sungkar et al. reported the same trend with *T. trichiura* being less sensitive to the same treatment protocol than *A. lumbricoides* and hookworm infection [31]. Concerning the other species, no change was observed in the incidence after intervention compared to before intervention. This suggests that the treatment of STH does not affect the spread of the disease in the population, probably because of an early re-exposure, but at least could contribute to the control of disease morbidity. We therefore think that to control STH in our context, acting on the risk factors is a necessity and will be the best approach to reduce the propagation of the disease, in combination with targeted treatment or large-scale treatment to reduce the STH morbidity in the population.

The follow-up of participants treated for STH during the second follow-up phase enabled us to assess the PTI rate and thus to estimate the potential impact of treatment on the presence of each STH species in the community. After 9 months of post-treatment follow-up, almost one participant out of two (44%, 95%CI: 31–58) was still infected with the same species of STH and could be considered reinfected or cases of treatment failure. Although with a longer period of follow-up, a similar infection rate post-treatment (35%, 95%CI: 27–42) and, in that case, clearly defined as reinfection was reported by Speich et al*.* in Tanzania among a cohort of school-aged children, 18 weeks after treatment [32]. Similarly, our finding is higher than the 7% infection rate found by Staudacher et al*.* among children in Rwanda, 3 months after treatment [24]. From our side, the level of the PTI rate could indicate that a part of our population is living in conditions exposing them to parasites, showing for them the necessity of health education with regard to STH infections. Indeed, the WHO recommends health education of the at-risk population to control the transmission of the disease in endemic areas [33]. We found *T. trichiura* as the STH species with the highest PTI rate. This result is consistent with the 37% PTI rate for *T*. *trichiura* reported by Speich et al. in Tanzania, and could indicate that *T. trichiura* should be a particular interest when fighting STH. Indeed, despite treatment, the prevalence of trichuriasis should continuously increase and become a major medical concern in endemic areas. Therefore, there is a need for an effective treatment of trichuriasis.

We found that gender and age were associated with being infected with STH parasites over the course of the study. Indeed, males were more likely to be infected with hookworm than female, while the risk of being infected with ascariasis decreased with age, in contrast to the risk of being infected with hookworm which increased with age. These results could indicate that gender and age-related behaviours influence the distribution of STH in the community. Similar results on gender as an associated factor for STH infections were reported in other studies [34, 35]. As suggested by Scott, we hypothesize that the risk of being infected with *A. lumbricoides* decreases in adulthood because adults care better about hygiene than children [35]. Similarly, we can assume that the increase in hookworm infection with age and its association with gender is because adults and particularly males could be more involved in activities exposing them to infested soil and hence hookworm infection. However, this needs to be further investigated.

### Limitations of the study

This study has some limitations. We used microscopy-based techniques for the diagnosis of STH infection. Although it is the goal standard method recommended by the WHO, the sensitivity of Kato-Katz remains low, particularly for light-intensity STH infections [36]. We indeed found, for instance, some participants negative for STH after being tested positive six months before and this without receiving any treatment. This situation probably led to misclassification bias but is not specific to our study. To improve the sensitivity of the technique, it is recommended to collect more than one stool sample, which was not the case in our study. However, as we used the same process at the three time-points of STH infection assessment, we remain confident our findings regarding the dynamic of STH infection in our community remain reliable. One of our main objectives was to determine the STH PTI rate in our cohort. As we did not assess the outcome of STH infection treatment, as another limitation of our study, we could not confirm the post-treatment cure. Therefore, the cases of infection observed post-treatment could be either re-infection, or a possibility of treatment failure, particularly for *T. trichiura* infection. Indeed, a previous study on the efficacy of ABZ for the treatment of STH infection conducted in the same area showed that one dose of ABZ per day for three consecutive days as we did, is more effective than a single dose which has a moderate effect on hookworm and *T. trichuriasis* infections [4]. These results make us more confident that most of those cases are reinfection cases. Furthermore, the main difficulty we encountered over the study course was to collect stool samples for each participant at the three time points of assessment, leading to missing stool samples for some participants at some assessment time points. Although this situation could affect the indicators of the presence of STH in our community we reported here, we remain confident that based on our sample size, our results reflect the situation of STH infection in the field.

## Conclusion

Our results confirm that the rural area around Lambaréné is endemic for STH infection and show that *T. trichiura* is the most prevalent species, indicating the necessity to re-think the drugs used in the community STH control programme as benzimidazole is known to be less effective. If the level of STH prevalence we reported here indicates the necessity to implement an annual deworming programme for the community to control disease morbidity our findings on the incidence highlight the need for the implementation of measures aiming to control the spread of the disease in the community. For tailored and effective control programmes, investigation of population practices related to STH infections and potential additional risk factors are needed to further explain the situation of STH infections in our community.

### Supplementary Information

**Additional file 1.** Classes of intensity for soil-transmitted helminth infections.

## Data Availability

The datasets used and/or analysed during the current study are available from the corresponding author on reasonable request.

## Abbreviations

ABZ: Albendazole
CERMEL: Centre de Recherches Médicales de Lambaréné
CI: Confidence interval
FU: Follow-up
FUP: Follow-up phase
OR: Odds ratio
PTI: Post-treatment infection
SD: Standard deviation
STH: Soil-transmitted helminth
WHO: World Health Organization
