# Supplementary material for: Epidemiology of soil-transmitted helminth infections and the differential effect of treatment on the distribution of helminth species in rural areas of Gabon
Source: Trop Med Health. 2024 Jan 2;52:3. doi: 10.1186/s41182-023-00567-z (PMC10759385; doi:10.1186/s41182-023-00567-z)
Supplement: Supplementary file 1 — Additional file 1. Classes of intensity for soil-transmitted helminth infections. [file 41182_2023_567_MOESM1_ESM.docx]

**Supplementary Table 1:** Classes of intensity for soil-transmitted helminth infections [1]

| Organism | Light-intensity infection | Moderate-intensity infection | Heavy-intensity infection |
| --- | --- | --- | --- |
| *Ascaris lumbricoides* | 1 – 4999 epg* | 5 000 – 49 999 epg | ≥ 50 000 epg |
| *Trichuris trichiura* | 1 – 999 epg | 1 000 – 9 999 epg | ≥ 10 000 epg |
| Hookworms | 1 – 1 999 epg | 2 000 – 3 999 epg | ≥ 4 000 epg |

*egg per gram

1. WHO Expert Committee on the Control of Schistosomiasis. Prevention and control of schistosomiasis and soil-transmitted helminthiasis: report of a WHO expert committee. WHO Technical Report Series; 912
